# Degenerative Disease Diagnosis and Analysis Based on Tissue Specificity of DNA Methylation

**DOI:** 10.3390/ijms26020452

**Published:** 2025-01-07

**Authors:** Jian Zhao, Wei Yao, Hanlin Gao, Zhejun Kuang, Lijuan Shi, Han Wang, Zhuozheng Dang

**Affiliations:** 1 School of Computer Science and Technology, Changchun University, Changchun 130022, China; zhaojian@ccu.edu.cn (J.Z.); 221501476@mails.ccu.edu.cn (W.Y.); 221501468@mails.ccu.edu.cn (H.G.); dangzhuozheng@163.com (Z.D.); 2 Jilin Provincial Key Laboratory of Human Health Status Identification Function & Enhancement, Changchun 130022, China; shilj@ccu.edu.cn; 3 Key Laboratory of Intelligent Rehabilitation and Barrier-Free for the Disabled, Changchun University, Ministry of Education, Changchun 130022, China; 4 College of Electronic Information Engineering, Changchun University, Changchun 130012, China; 5 School of Information Science and Technology, Institute of Computational Biology, Northeast Normal University, Changchun 130117, China

**Keywords:** DNA methylation, tissue specificity, disease specificity, CpG site, chi-square analysis, logistic regression, transformer model

## Abstract

The tissue specificity of DNA methylation refers to the significant differences in DNA methylation patterns in different tissues. This specificity regulates gene expression, thereby supporting the specific functions of each tissue and the maintenance of normal physiological activities. Abnormal tissue-specific patterns of DNA methylation are closely related to age-related diseases. This abnormal methylation pattern affects the regulation of gene expression, which may lead to changes in cell function and promote the occurrence of pathological conditions. By analyzing the differences in these methylation patterns, key CpG sites for disease diagnosis can be effectively screened. The main goal of this paper is to use the characteristics associated with tissue-specific abnormal expression and disease to construct an age-related disease diagnosis model. First, we combined chi-square tests and logistic regression to identify tissue-specific and disease-specific CpG sites, laying the foundation for accurate medical diagnosis, and verified the biological relevance of these CpG sites through enrichment analysis. Then we used the Transformer model to fit these CpG sites and realized the automatic diagnosis of age-related diseases. Our work proves that the tissue specificity of DNA methylation has the potential to diagnose age-related diseases, and proves the scientific nature of our proposed diagnostic method from a biological perspective.

## 1. Introduction

Age-Related Diseases refers to a class of diseases whose risk of onset gradually increases with age, including a variety of degenerative diseases and cancers. Such diseases involve complex pathological mechanisms, such as cell aging and tissue function degeneration, which seriously affect the health and quality of life of the elderly [1]. Degenerative diseases are a class of diseases that involve the gradual loss of tissue and organ function, such as Alzheimer’s disease and Parkinson’s disease. They are usually related to the aging process of the body. As the population ages, their incidence and social burden are increasing [2,3]. Changes in DNA methylation contribute to the pathology of age-related diseases [4]. Therefore, it is crucial to study age-related Diseases using the pattern of DNA methylation.

DNA epigenetics refers to a mechanism that regulates gene expression through reversible modification without changing the DNA sequence. It mainly occurs on cytosine (C) in CpG islands, affecting the accessibility and activity of gene expression, thereby controlling the functional differentiation of cells [5]. DNA methylation tissue specificity refers to the differences in the degree of methylation in different tissues. This difference represents different cell functions and maintains tissue characteristics [6]. Aging affects DNA methylation in a tissue-specific manner, and such changes have been implicated in a variety of age-related diseases [7]. Therefore, we can diagnose age-related diseases by analyzing the tissue-specific patterns of DNA methylation.

With the rapid development of artificial intelligence technology, advanced technologies such as machine learning have become important tools for DNA methylation data research and are widely used in the analysis of methylation data to help researchers predict disease risks and diagnose conditions. Previous work mainly compared the methylation patterns of normal tissues and corresponding disease tissues, identified disease-specific methylation changes, and used machine learning models to diagnose diseases [8,9,10]. These studies have achieved good results in disease diagnosis, which shows that machine learning technology has great potential in the task of automatically diagnosing diseases using DNA methylation data. In recent years, deep learning has also been widely used in the task of disease diagnosis using DNA methylation data. (Park et al.) [11] used Multilayer Perceptron (MLP) to achieve automated diagnosis of Alzheimer’s disease. (Liu et al.) [12] used Artificial Neuron Network(ANN) to diagnose cancer. However, these studies focused on the numerical differences in DNA methylation patterns between healthy samples and normal samples in the process of screening key CpG sites, rather than finding key CpG sites that are truly diagnostically significant.

This study takes advantage of the fact that abnormal tissue-specific DNA methylation patterns are closely related to age-related diseases and proposes an innovative diagnostic method for neurodegenerative diseases and multiple cancers. Unlike previous studies that only compared the numerical differences in DNA methylation patterns between normal and diseased tissues or only focused on the changes in single CpG sites, this study combines tissue-specific and disease-specific methylation patterns to explore the synergistic mechanism of methylation patterns that promote age-related diseases, thereby screening out CpG sites that are truly diagnostically significant, and verifies the scientific nature of this diagnostic method from a biological perspective through enrichment analysis. In order to better utilize the synergistic mechanism between these key CpG sites, we developed a Transformer-based model combined with a dynamic residual mechanism, which can capture the complex nonlinear relationship between CpG sites and enhance the model’s ability to focus on key features. This method not only improves the accuracy and efficiency of age-ralated disease classification, but also provides new insights into the role of tissue-specific DNA methylation in disease diagnosis. The uniqueness of this study is that it incorporates tissue-specific DNA methylation abnormalities into the diagnostic process, which provides new perspectives and advanced methods compared to other existing studies in this field, and is of great significance for optimizing diagnostic strategies and advancing the study of age-related diseases.

## 2. Results

### 2.1. Tissue-Specific Site Validation

**Figure 1:**
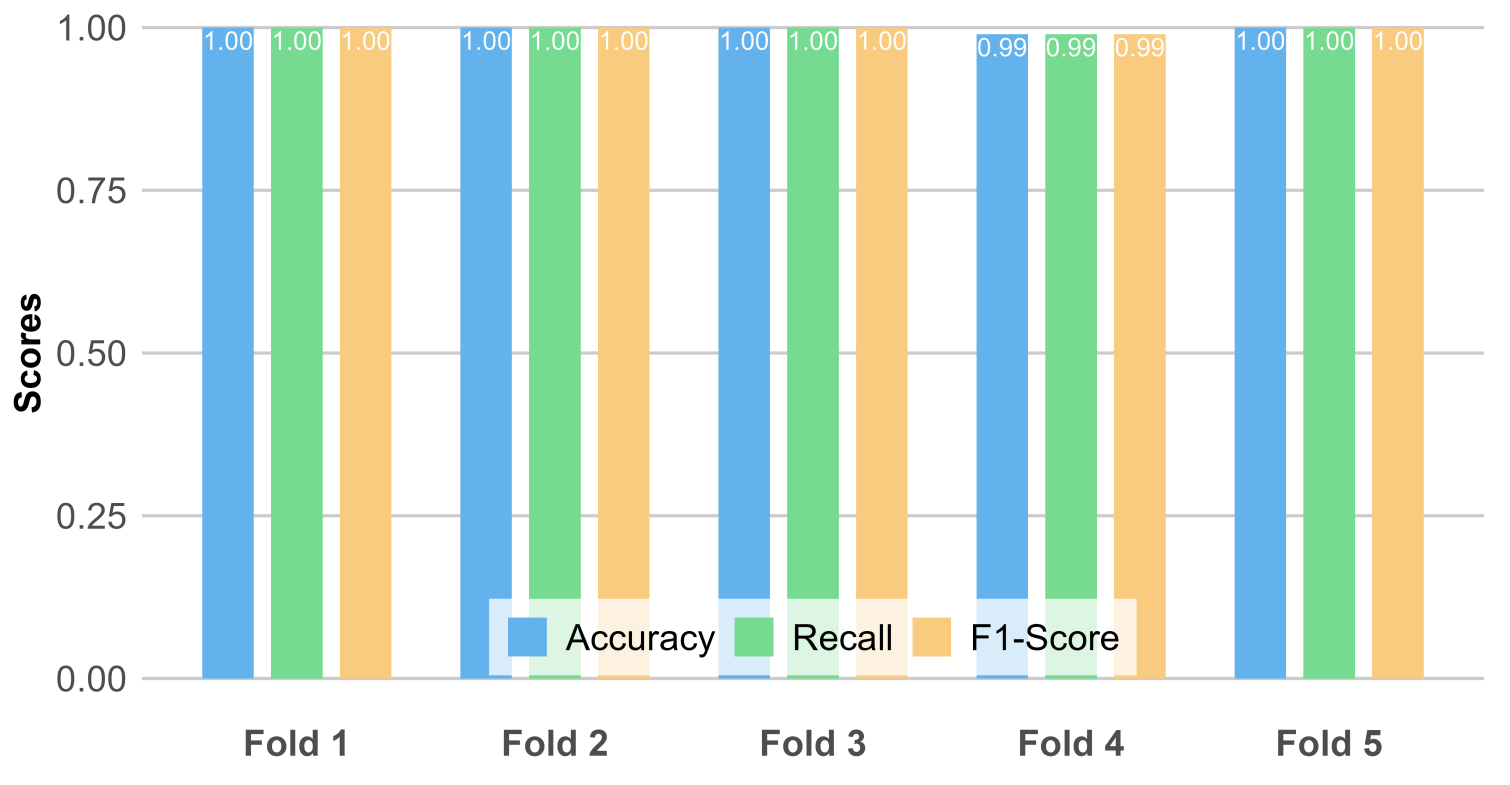
Five-fold cross-validation results for tissue classification based on selected CpG sites. This bar chart presents the performance metrics—Accuracy, Recall, and F1-Score—across five cross-validation folds. These results validate the tissue specificity of the CpG sites post-selection.

Initially, we utilized chi-square analysis to identify CpG sites potentially associated with specific tissue types from a large-scale dataset. Subsequently, these sites were used as features to predict the tissue type of samples using a logistic regression model, thereby verifying the biological and statistical significance of these CpG sites. This experiment aims to confirm the tissue-specific sites identified by chi-square analysis in terms of accuracy. To more precisely evaluate the model’s performance, we employed a five-fold cross-validation method. Specifically, the data were divided into five parts; four were used sequentially for model training, and the remaining one served for testing. This approach allows us to calculate the model’s average prediction accuracy across different numbers of CpG sites, effectively reducing the impact of random factors on the experimental results and providing a stable and reliable performance evaluation. To visually represent this process and the results, we utilized a bar chart to record the accuracy of each cross-validation iteration, as shown in the Figure 1. It displays three evaluative metrics: accuracy, recall, and F1 score, across five distinct folds. The results indicate that the first, second, third, and fifth folds achieved a perfect score of 1.0 across all metrics, whereas the fourth fold experienced a slight reduction in each metric to 0.99. These highly consistent scores substantiate that the CpG sites we identified are indeed distinctly tissue-specific.

### 2.2. Disease-Specific Site Validation

**Figure 2:**
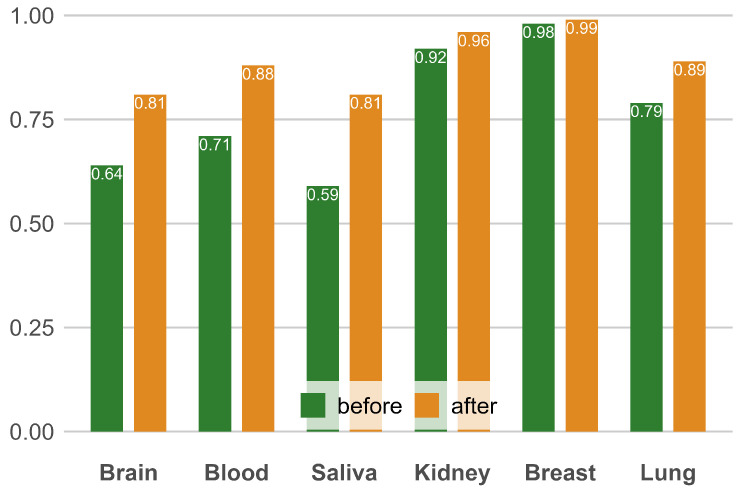
Comparison of classification accuracy of different tissue types before and after screening disease-specific CpG sites. This figure shows the classification accuracy of each tissue before screening (green) and after screening (orange). The comparison proves that the CpG sites we screened are specific diseases.

This study employed a logistic regression model to screen for disease-specific CpG sites from tissue-specific sites, using health status as the label. Through this approach, we effectively identified a set of CpG sites that are both tissue-specific and disease-specific. To ensure the stability and reliability of the evaluation results, we conducted a five-fold cross-validation. For each tissue type, we independently developed a predictive model and compared the results before and after screening. Additionally, we utilized a bar chart to intuitively display these comparative results. As shown in the Figure 2, after CpG site selection, the prediction accuracy for most tissues improved. Specifically, after site selection for the kidney, saliva, and blood, the median accuracy increased significantly, while the accuracy for the breast and brain did not improve as much. This is because the model using all sites had already achieved high accuracy, leaving limited room for further optimization. In summary, the CpG sites selected by this work are both tissue-specific and disease-specific.

In order to reflect the differences in methylation levels of specific CpG sites in healthy and diseased states, this article lists the median of each site in healthy and diseased states, namely Median of healthy samples (H_M), Median of disease samples (D_M) and the magnification (D_M/H_M) and standard error, respectively Standard error for healthy samples (H_SE) and Standard error of disease samples (D_SE), to show disease-related methylation changes. As shown in Table 1. For full CpG site information, please see Supplementary Materials.

### 2.3. Performance of the Diagnostic Model

In order to evaluate and verify the ability of our model to diagnose age-related diseases in different biological tissues (breast, kidney, lung, saliva, blood, and brain) and the effectiveness of the dynamic residual module, we trained and evaluated multiple machine learning models including Logistic Regression, Support Vector Machine (SVM), random forest, XGBoost and K-Nearest Neighbor (KNN) as well as our proposed model through a five-fold cross-validation method to ensure that each model can be fully tested on different tissue datasets. The baseline model refers to the model without the dynamic residual module. This study effectively demonstrated the diagnostic performance of each model by drawing and comparing the Receiver Operating Characteristic (ROC) curves and Area Under the Curve (AUC) values of each model on each tissue dataset, and verified the effect of the dynamic residual module in improving the diagnostic ability of the model.

**Figure 3:**
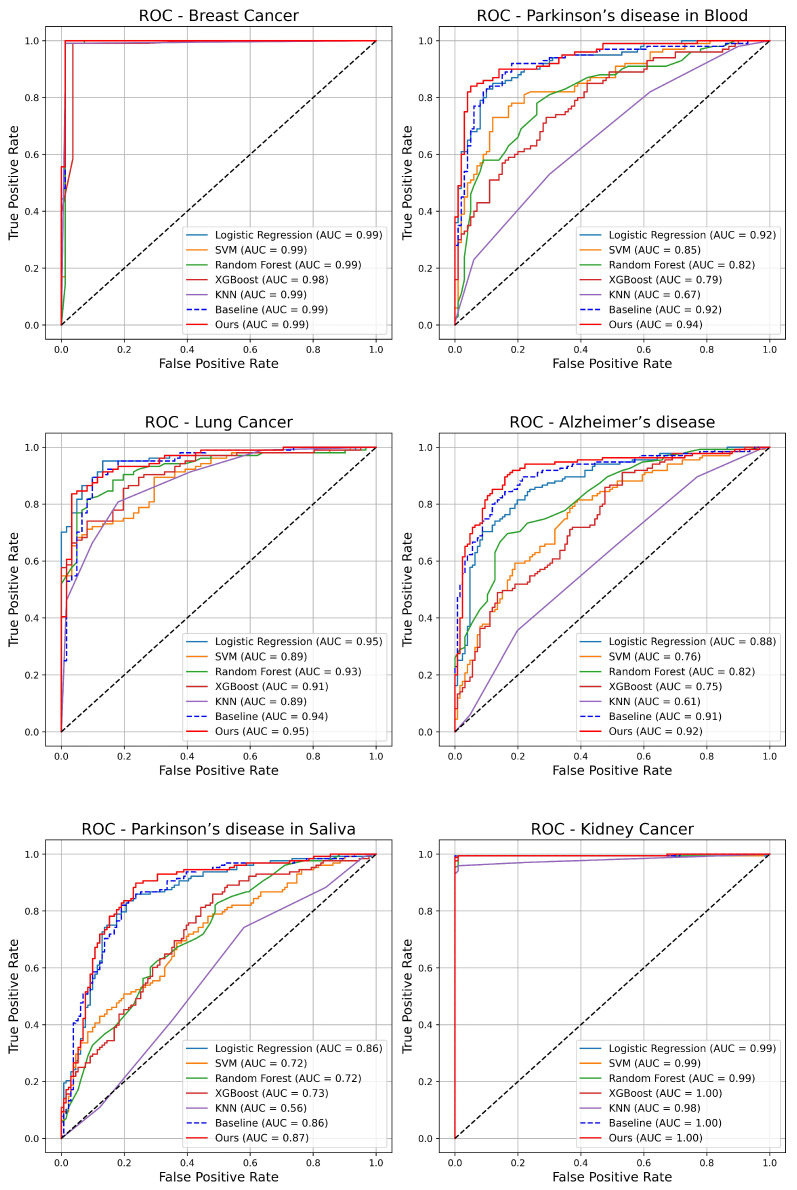
The ROC curve shows the performance comparison of our model with other commonly used machine learning models and baseline models on various tissues. Each figure shows the relationship between the true positive rate (TPR) and false positive rate (FPR) of different models on specific tissue samples. The AUC value of each model is proportional to its ability to diagnose the disease. These figures can intuitively compare the diagnostic ability of different models for the disease. The red curve is our model.

As can be seen from Figure 3, our model achieved a result close to 1.0 in breast and kidney, which means that our model can diagnose breast cancer and kidney cancer very well. In blood, brain, and lung tissues, our model’s diagnostic effect is better than that of traditional machine learning models, and the model with the dynamic residual module is better than the model without the dynamic residual module. In saliva tissue, our model achieved an AUC value of 0.87, which is better than other models, but compared with other tissues, it still has room for improvement.

### 2.4. Independent Testing

In order to test the performance of the model in a real clinical setting, this study designed an independent test experiment. We first trained four models on lung, brain, whole blood, and breast tissues using selected CpG sites as features, using all the data analyzed above as training sets. Then, to test the performance of these models when processing unknown data, this paper selected four datasets of the same type as the training set but completely independent for testing. Such independent tests not only evaluate the performance of the model on new data, but also demonstrate the stability and reliability of the model when processing similar data in actual clinical applications.

In independent tests of four tissue types, our model achieved an accuracy of 0.96 on breast and lung tissue samples, showing high reliability and sensitivity. For brain tissue, the model’s accuracy was 0.97, showing its strong ability to identify Alzheimer’s disease in brain samples, providing substantial evidence for its potential application in clinical neuropathology diagnosis. However, for whole blood samples, the model’s accuracy was 0.71, reaching a moderate level, indicating that there is still room for improvement in performance on blood samples.

### 2.5. Enrichment Analysis

In our previous work, this paper used a combination of mathematical statistics and machine learning to screen out CpG sites in various tissues that are both tissue-specific and disease-specific, and used the Metaspace platform to perform GO (Gene Ontology) enrichment analysis and KEGG enrichment analysis on the genes corresponding to these CpG sites, revealing the concentrated expression of these genes in specific biological functions and their concentrated effects in metabolic pathways and biological signaling pathways, and at the same time verifying the rationality of the sites we screened from a biological perspective. In the figure, -log represents the correlation between the sites we screened and the pathways. The larger the value, the higher the correlation and the darker the color. Some of the CpG sites we screened and their corresponding genes are shown in Table 2. For full CpG site information, please see Supplementary Materials.

#### 2.5.1. Enrichment Analysis of Brain

**Figure 4:**
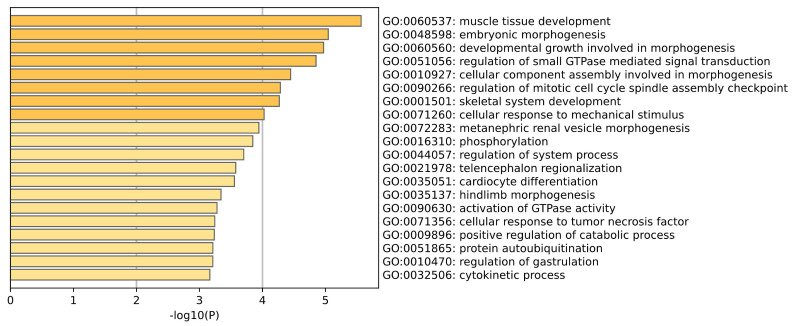
This plot is a GO enrichment analysis of Brain tissue.

GO enrichment analysis of brain tissue is shown in the Figure 4. Among the tissue–specific and Alzheimer’s disease–specific genes screened in brain tissue, the biological processes we observed showed a series of biological processes closely related to brain development and function. Phosphorylation is a key way to regulate protein function and signal transduction pathways, and its abnormality may lead to imbalances in multiple neural pathways. (Ferrer et al.) [13] explored the disorder of protein phosphorylation in brain aging and various stages of AD, pointing out that disordered phosphorylated proteins can affect synaptic transmission and membrane signal transduction, suggesting that they may be an early indicator of mild cognitive impairment. GTPase plays an important role in regulating intracellular transport, cytoskeleton dynamics and cell proliferation, and their disorder can lead to abnormalities in neural pathways. (Stankiewicz et al.) [14] pointed out that Rho family GTPases play a key role in neuronal morphology and survival, among which Rac GTPase promotes neurite growth and neuronal survival, while Rho GTPase triggers neurite retraction and neuronal apoptosis. (Rajaei et al.) [15] studied the conformational changes and GTPase activity of tubulin in the brain tissue of Alzheimer’s disease patients and found that the GTPase activity in AD patient samples was significantly higher than that in healthy samples.

**Figure 5:**
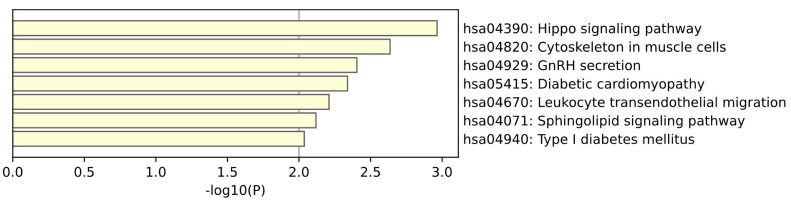
This plot is a KEGG enrichment analysis of Brain tissue.

KEGG enrichment analysis of brain tissue is shown in the Figure 5. Among the sites that are both tissue-specific and disease-specific, the pathways enriched in brain tissue revealed multiple signaling pathways that may be associated with disease progression. The sphingomyelin signaling pathway plays a key role in regulating cell survival, proliferation, migration, and apoptosis. Sphingomyelin and its metabolites such as ceramide can transmit signals through multiple signaling pathways, affecting cell fate and function. In the nervous system, these molecules are involved in regulating inflammatory responses, neuroprotection, and cell death, and are associated with the occurrence and development of a variety of neurodegenerative diseases. In brain diseases, abnormal leukocyte migration may be associated with neuroinflammation and neurodegeneration. (Mühle et al.) [16] studied the role of phospholipid synthases in neuropsychiatric health and disease. Sphingomyelin and its metabolites are involved in multiple signaling pathways that regulate neuronal survival, proliferation, and differentiation. The Hippo signaling pathway is an important pathway for regulating organ size, cell proliferation and apoptosis, and controls cell growth by inhibiting the activity of YAP and TAZ. (Wei et al.) [17] studied the role of the Hippo signaling pathway in ischemia-related central nervous system diseases and concluded that the Hippo pathway plays an important role in the pathogenesis of AD by affecting oxidative stress, inflammatory response, blood–brain barrier integrity, mitochondrial dysfunction and neuronal cell death.

#### 2.5.2. Enrichment Analysis of Kidney

**Figure 6:**
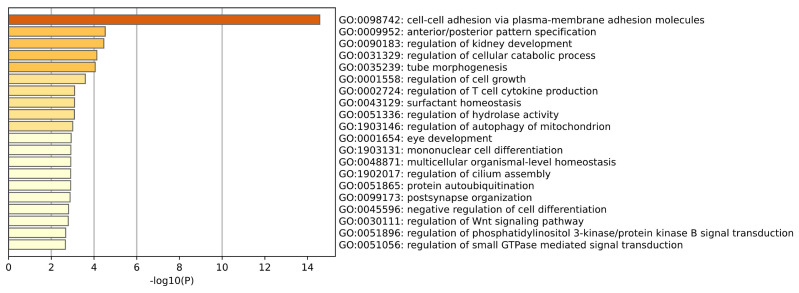
This plot is a GO enrichment analysis of Kidney tissue.

GO enrichment analysis of Kidney tissue is shown in the Figure 6. As can be seen from the figure above, in the gene ontology (GO) enrichment analysis of this kidney tissue, the regulation of renal development, tubular formation, and surfactant homeostasis directly reflect the basic functions of the kidney, involving the formation, maintenance, and regulation of filtration function of renal tubules. The positive regulation of cell proliferation, the regulation of the Wnt signaling pathway, the transfer factor signaling pathway, and the process of cell migration are related to the characteristics of renal cancer, highlighting the key links in the occurrence and development of renal cancer, such as imbalance in cell cycle regulation, abnormal signal transduction, and changes in cell behavior. (Wang et al.) [18] summarized the role of the Wnt signaling pathway in kidney development and showed that its dysregulated expression can lead to developmental abnormalities and kidney diseases such as congenital kidney, cystic kidney and renal cancer.

**Figure 7:**
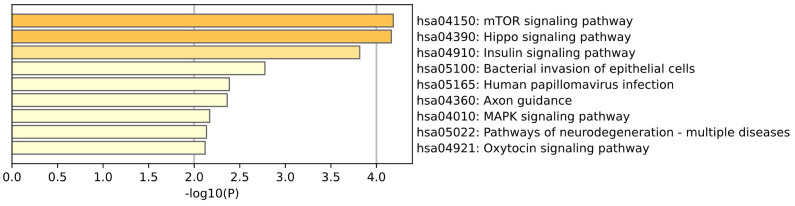
This plot is a KEGG enrichment analysis of kidney tissue.

KEGG enrichment analysis of Kidney tissue is shown in the Figure 7. The mTOR signaling pathway is notable for its central role in regulating cell growth, metabolism, and autophagy, and is closely related to the proliferation and survival of renal cancer cells. (Satardey et al.) [19] analyzed the expression patterns of AKT and HIF-1 in the AKT/mTOR signaling pathway and their prognostic significance and found that these proteins were highly expressed in renal cancer tissues. Similarly, the MAPK signaling pathway also plays a key regulatory role in cell proliferation and differentiation, and its abnormal activation in renal cancer is directly related to the formation and progression of tumors. (Borelli et al.) [20] found that inhibiting one or more MAPK signaling pathways can inhibit the growth of renal cell carcinoma by destroying the tumor vascular structure. This study provides a theoretical basis for developing new treatment strategies for renal cell carcinoma. The Hippo signaling pathway (hsa04390) has been linked to the regulation of cell growth and organ size, and its role in kidney development may be reflected in the dysregulation of growth in renal cancer cells. (Cinar et al.) [21] summarized the role of the Hippo pathway in prostate cancer, renal cancer and bladder cancer. Its core kinases MST1/2 and LATS1/2 regulate this pathway in mammals by phosphorylating and inactivating YAP1 signaling. In renal cancer cells, the function of these kinases is often lost, leading to the overactivation of YAP1 and TAZ. This activation promotes tumor cell proliferation and metastasis. The insulin signaling pathway plays a role in maintaining blood glucose homeostasis and regulating renal filtration function, while the oxytocin signaling pathway may affect the renal water reabsorption mechanism and blood pressure regulation. (Solarek et al.) [22] found that insulin plays a stimulatory role in the growth and migration of RCC cells. Although the expression of insulin receptor (IR) in RCC cells was downregulated, these cells still responded to insulin stimulation through IGF1R.

#### 2.5.3. Enrichment Analysis of Saliva

**Figure 8:**
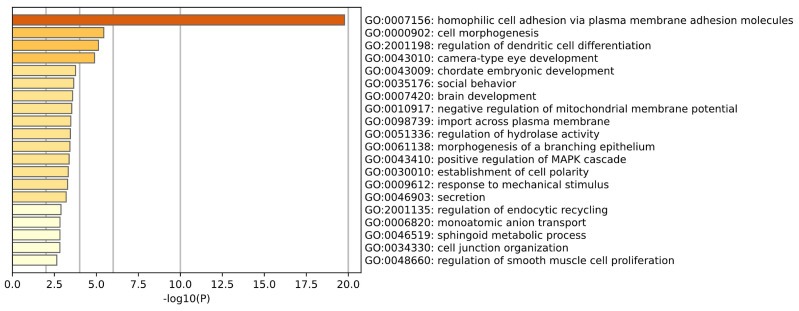
This plot is a GO enrichment analysis of Saliva tissue.

GO enrichment analysis of Saliva tissue is shown in the Figure 8. In the GO enrichment analysis bar chart above, secretion and homogeneous cell adhesion are two biological processes directly related to the physiological activities and cell-to-cell interactions of the salivary glands. Secretion is related to the basic functions of the salivary glands, and homogeneous cell adhesion affects the stability and structural organization of cells in the salivary glands. (Figura et al.) [23] By studying saliva samples from Parkinson’s patients, it was found that the salivary proteome composition of PD patients was different from that of healthy controls, with lower concentrations of proteins involved in the inflammatory process, exosome formation, and adipose tissue formation. Brain development and negative regulation of mitochondrial membrane potential are associated with Parkinson’s disease. Impaired brain development may be related to the pathological mechanism of Parkinson’s disease. Changes in mitochondrial function are associated with the development of neurodegenerative diseases and may be related to the abnormal energy metabolism and oxidative stress observed in the disease. (Kai Yu et al.) [24] The study found that the Parkinson’s-related genes PINK1 and PINK2 are directly involved in processes such as mitophagy that maintain mitochondrial health.

**Figure 9:**
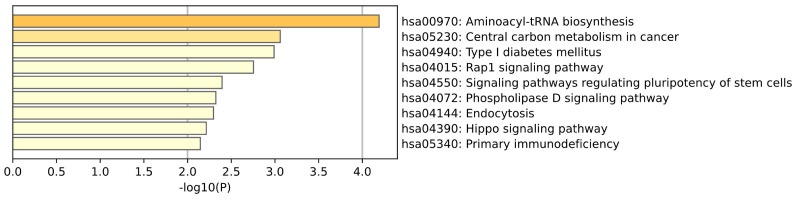
This plot is a KEGG enrichment analysis of Saliva tissue.

KEGG enrichment analysis of Saliva tissue is shown in the Figure 9. Pathways of aminoacyl-tRNA biosynthesis, Rap1 signaling, phospholipase D signaling, and endocytosis may be associated with metabolic stress in neurons, disturbances in neural signaling, and neurotransmitter processing, which reflects the molecular mechanism of neurodegeneration in Parkinson’s disease. (Corti et al.) [25] found that the p38 subunit is a substrate of Parkin and that ubiquitination of p38 was abolished by a truncated variant of Parkin lacking the essential functional domain, whereas p38 is a key structural component of the mammalian aminoacyl–tRNA synthetase complex. (Stieglitz et al.) [26] summarized the drugs developed for phospholipase D and proposed that human phospholipase D is not only a therapeutic target for diseases such as cardiovascular disease and cancer, but also a therapeutic target for neurodegenerative diseases including Alzheimer’s disease and Parkinson’s disease.

#### 2.5.4. Enrichment Analysis of Whole Blood

**Figure 10:**
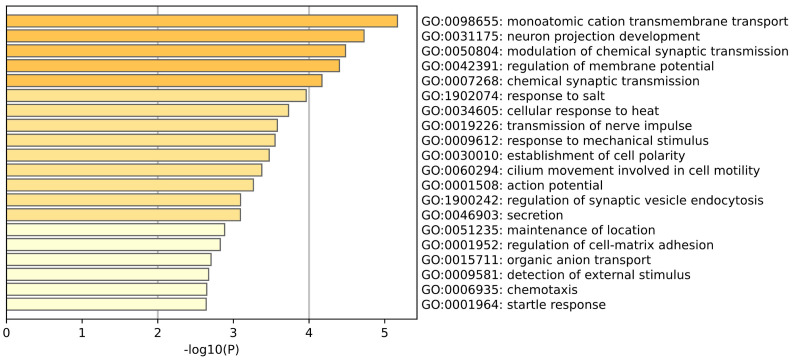
This plot is a GO enrichment analysis of Whole Blood tissue.

GO enrichment analysis of Whole Blood tissue is shown in the Figure 10. Through gene ontology analysis of blood samples, we identified multiple biological processes associated with the physiological functions of blood and Parkinson’s disease. Those related to blood include maintenance of position, secretion, transport of organic anions and transmembrane transport of single atomic cations. Maintenance of position is related to the distribution and positioning of blood cells in the circulation. The secretion of molecules involves the release of hormones and other important molecules in the blood. The transport of organic anions involves the removal of metabolic waste and the regulation of nutrient delivery. Transmembrane transport of single atomic cations plays an important role in maintaining blood electrolyte balance. The normal operation of these processes is essential for maintaining blood biochemical homeostasis. The biological processes associated with Parkinson’s disease include neuronal projection, synaptic transmission, regulation of membrane potential, and regulation of synaptic vesicle endocytosis. The development of neuronal projection is the basis for the formation of neural communication networks, involving communication and connections between neurons, which may be impaired in PD, leading to the decline of cognitive and motor functions. Synaptic transmission involves the transmission of neurotransmitters between synapses. PD patients are impaired in this process, especially affecting the transmission of dopamine, which is the core mechanism leading to motor and non–motor symptoms. (Stern et al.) [27] By comparing Parkinson’s patients and healthy samples, it was found that the synaptic current rate of neurons in PD patients was severely reduced. The regulation of membrane potential directly affects the activation state of neurons, and its disorder in PD can further aggravate neurological dysfunction; the regulation of synaptic vesicle endocytosis involves the reabsorption and reuse of neurotransmitters by nerve cells. During the course of PD, the reuse of neurotransmitter circulation may be affected, thereby affecting the efficiency and accuracy of neurotransmission. (Qadri et al.) [28] Studies have found that the mitochondrial membrane potential of PD patients is significantly reduced, so the mitochondrial membrane potential of peripheral blood mononuclear cells can be used as a marker for the diagnosis of Parkinson’s disease.

**Figure 11:**
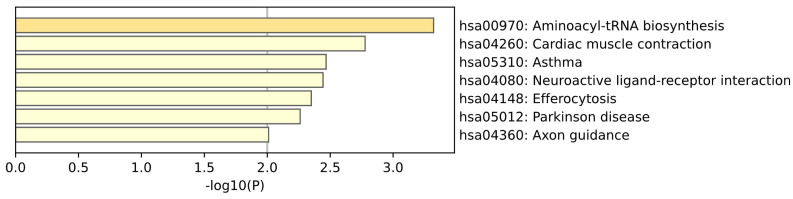
This plot is a KEGG enrichment analysis of Whole Blood tissue.

KEGG enrichment analysis of Whole Blood tissue is shown in the Figure 11. The gene pathways mapped to the analysis of whole blood tissue showed that we also enriched aminoacyl–tRNA biosynthesis in blood tissue, which illustrates its important role in Parkinson’s disease. Neuroactive ligand–receptor interaction pathways and PD–specific pathways are directly related to neurotransmitter imbalance and neuroinflammation in the disease. (Yan Kong et al.) [29] used high-throughput small RNA sequencing technology to study the miRNA expression of α–synuclein and found that the expression of these miRNAs significantly affects the neuroactive ligand–receptor interaction pathway.

#### 2.5.5. Enrichment Analysis of Lung

**Figure 12:**
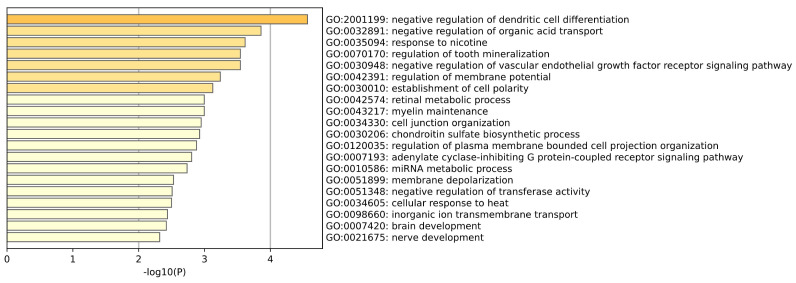
This plot is a GO enrichment analysis of lung tissue.

GO enrichment analysis of Lung tissue is shown in the Figure 12. Through gene ontology analysis of lung samples, we can see the physiological role of the lung in regulating transmembrane transport of inorganic ions, responding to temperature changes, and maintaining cell connections and extracellular matrix composition, reflecting its core function in gas exchange and environmental adaptation. There are also some biological processes associated with the development of lung cancer, such as membrane depolarization, regulation of transferase activity, inhibition of angiogenic signaling pathways, and changes in dendritic cell function in the immune response. These biological events reflect the adaptive mechanisms of tumor cells in proliferation, vascular invasion and escape from immune surveillance. (Tang et al.) [30] found that the expression of glutathione S-transferase M2 (GST-M2) was silenced by the hypermethylation binding of specific protein 1 (Sp1) in lung cancer cells, which may lead to the reduction of GST-M2 expression in lung cancer cells. (Li et al.) [31] analyzed the application and research status of angiogenesis inhibitors in the treatment of lung cancer, and pointed out that the current anti-angiogenesis drugs targeting VEGF or receptor tyrosine kinase have a certain effect in the treatment of lung cancer.

**Figure 13:**
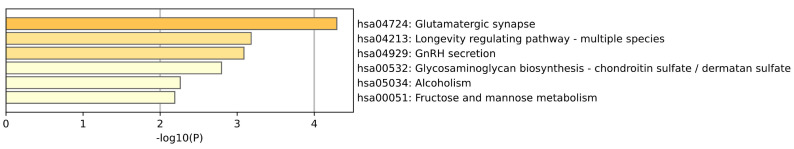
This plot is a KEGG enrichment analysis of lung tissue.

KEGG enrichment analysis of Lung tissue is shown in the Figure 13. From the enriched pathways in the figure, we can see that in lung tissue, the glycosaminoglycan biosynthesis pathway is essential for building lung extracellular matrix and regulating lung function. (Jenny Wigén et al.) [32] pointed out that glycosaminoglycans regulate cell activities by binding growth factors and morphogenetic factors and play an important role in cell differentiation and tissue regeneration. At the same time, the development of lung cancer may be closely related to the cellular anti-aging mechanisms and regulation of tumor metabolism in lifespan regulation pathways, as well as changes in energy metabolism and biosynthesis requirements in fructose and mannose metabolic pathways. (Krause et al.) [33] points out that fructose metabolism provides a carbon source for nucleotide synthesis, supports the synthesis of DNA and RNA, and promotes the rapid proliferation of cancer cells.

#### 2.5.6. Enrichment Analysis of Breast

**Figure 14:**
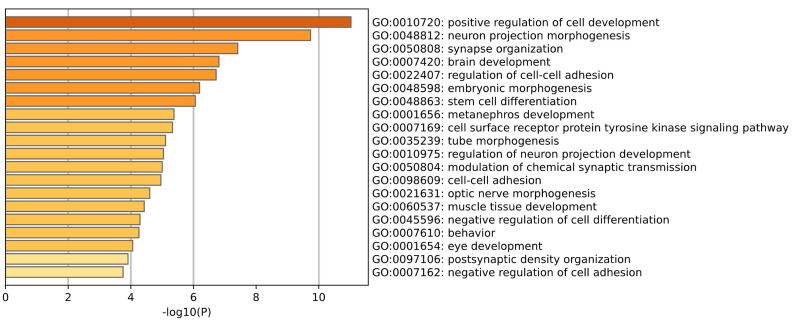
This plot is a GO enrichment analysis of Brease tissue.

GO enrichment analysis of Breast tissue is shown in the Figure 14. Gene ontology analysis of breast tissue samples revealed biological processes associated with normal growth, development, and functional maintenance of the mammary gland, including cell differentiation, tissue morphogenesis, and growth regulation. There are also some biological processes that may play a key role in the development of breast cancer, including cell adhesion, migration ability, and regulation of kinase signaling pathways, which play a role in the proliferation, invasion, and metastasis of breast cancer cells. In particular, integrin-mediated regulation of cell adhesion and protein tyrosine kinase signaling pathways are key factors in regulating tumor growth and metastatic potential. (Menashe et al.) [34] conducted an in-depth analysis of the whole genome association study of breast cancer and found that the pathways of key components of the protein kinase signaling cascade are very important in breast cancer susceptibility.

**Figure 15:**
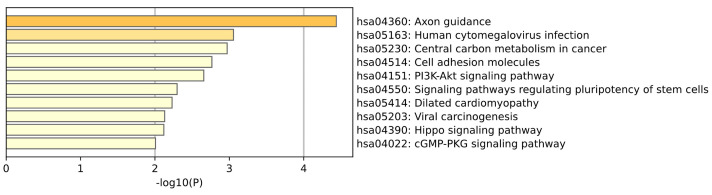
This plot is a KEGG enrichment analysis of Breast tissue.

KEGG enrichment analysis of Breast tissue is shown in the Figure 15. Pathways directly related to breast cancer include central carbon metabolism, cell adhesion molecules, PI3K-Akt signaling pathway, stem cell pluripotency regulation, viral carcinogenesis, and Hippo signaling pathway, which play a core role in tumor energy metabolism, cell-cell interaction, signal transduction, stem cell characteristics, and tumor growth regulation. Breast-related pathways may involve regulatory pathways for stem cell pluripotency, which is essential for the development and maintenance of breast tissue. (Sun et al.) [35] found that the PI3K-AKT signaling pathway is overactivated in breast cancer. AKT phosphorylates and inhibits salt-induced kinase 1 (SIK1), thereby relieving its inhibition on STAT3 and promoting the occurrence of breast tumors.

## 3. Discussion

This study used a method combining mathematical statistics with machine learning to screen out key CpG sites with diagnostic significance at the whole genome level to ensure that the selected sites are closely related to relevant tissues and age-related diseases, providing a basis for the development of subsequent diagnostic models. In order to better fit these sites, we used a Transformer-based method combined with a dynamic residual mechanism to establish a model that can accurately diagnose specific diseases. The model showed good performance and generalization ability on the test set, proving its effectiveness in practical medical applications. The scientific nature of the diagnostic method proposed in this article was verified from a biological perspective through GO and KEGG enrichment analysis.

First, this paper used the chi-square test to identify highly significant tissue-specific CpG sites. Subsequently, we screened these CpG sites for those associated with specific age-ralated diseases through logistic regression analysis to ensure that the selected sites were both tissue-specific and disease-specific. (Chad et al.) [9] used an unsupervised clustering method to study the methylation variability of gene promoter regions, revealing the most stable promoter regions in specific tissues, which are often associated with genes that are indispensable for tissue function. However, their study focused on methylation variability within gene promoter regions rather than individual CpG sites, which may overlook subtle changes at the CpG site level that may have predictive value for disease. In contrast, our method targets specific CpG sites and can identify and analyze subtle methylation changes that may be highly associated with specific diseases. (Emilie et al.) [36] studied the tissue specificity of DNA methylation in neonatal and placental tissues, emphasizing its significance for future epigenetic epidemiological studies. They used Fisher’s exact probability method to identify methylation regions associated with cell function, movement, signal transduction, immune response, and embryonic development. This method is suitable for the analysis of small data sets, but when the sample size is large, the method becomes complicated and time-consuming. Machine learning technology is good at processing more complex data sets and patterns, improving the efficiency of extracting useful information from large-scale epigenetic data, and achieving effective feature selection, thereby improving the predictive performance and biological interpretability of the analysis model. (Yan-Zhe Wang et al.) [37] found that changes in the methylation status of core regulatory genes may be a potential factor in the onset of diabetic nephropathy, providing potential molecular targets for early diagnosis and treatment strategies. Although their study recognized differentially methylated sites, it mainly relied on comparing known tissue-specific patterns and may not have fully utilized machine learning technology to predict the complex relationship between unknown, potentially important methylation sites and disease states. To overcome the limitations of previous studies, This paper specifically utilized the high sensitivity of the chi-square test, which is an ideal tool for determining significant associations between the methylation status of CpG sites and specific tissue types. This approach enables us to accurately capture tissue-specific sites, laying a solid foundation for subsequent in-depth analysis. In addition, logistic regression is known for its strong predictive power and high output interpretability. It not only generates prediction results but also clarifies the strength and direction of the impact of each variable on the prediction through regression coefficients, enhancing our understanding of which CpG sites play a key role in disease development.

The tissue-specific sites we identified showed a performance close to 1.0 in the logistic regression model, indicating that the sites we screened were tissue-specific. Further analysis found that the disease-specific CpG sites further screened by logistic regression had higher accuracy in various tissues through the verification of logistic regression, indicating that the sites we screened were also disease-specific. We fitted these sites with a age-ralated disease diagnosis model and obtained a better AUC value than the traditional machine learning model, indicating that our model can achieve better diagnostic results. This paper then located these CpG sites on specific genes and performed GO and KEGG enrichment analysis to find biological processes, pathways, or functions that were significantly enriched at these sites, thereby clarifying the biological significance behind the changes in gene expression. The main biological processes identified in this study include stem cell differentiation, cell adhesion, gap junctions, and signal transduction pathways such as TGF-β, Hippo, and PI3K-Akt, which are critical for cell function and age-ralated disease progression. Abnormalities in stem cell pathways may lead to neuronal degeneration, while disruption of cell adhesion and gap junctions is associated with neuronal loss. Notably, aberrant activation or inhibition of these pathways plays a key role in the pathogenesis of Alzheimer’s disease and various cancers, including renal and breast cancer. In addition, alterations in central carbon metabolism and energy metabolism highlight their importance in disease states, reflecting dysregulation of metabolic waste disposal and energy balance. The results of enrichment analysis indicate that our proposed diagnostic approach can not only achieve good results but also has scientific validity from a biological perspective.

Although our method has achieved good results in the task of disease diagnosis, it still faces some shortcomings. In the task of diagnosing Parkinson’s disease in salivary tissue, although our model also achieved an AUC value of 0.87, there is still room for improvement compared with our diagnostic model in other tissues. This may be because saliva samples are not as sensitive to diseases as other tissues. We will improve the model to better capture subtle changes in the disease. In experiments where blood samples were independently tested to verify the generalization ability of our model, our model’s performance in blood was not as good as other tissues, which shows that there is room for further optimization of the generalization ability of our model in blood samples. In the future, we will use better technology to improve the generalization ability of the model in blood tissue.

## 4. Materials and Methods

### 4.1. Workflow

**Figure 16:**
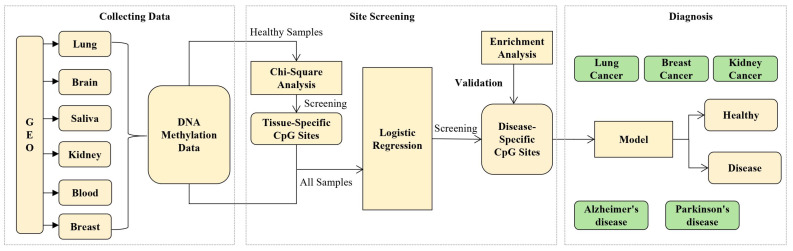
This figure illustrates the research method framework used in our paper. It shows that starting from the DNA methylation data of multiple tissues, through chi-square analysis and logistic regression, CpG sites associated with tissues and diseases are identified, and the correctness of these sites is verified by enrichment analysis, and a diagnostic model that can distinguish between healthy and disease states is constructed.

The research method flow of this paper is shown in Figure 16. This study first obtained DNA methylation data from the Gene Expression Omnibus (GEO) database and determined tissue-specific CpG sites through chi-square analysis. Then, the logistic regression method was used to further screen out key CpG sites that can accurately diagnose age-related diseases, and these sites were enriched and analyzed to prove the correctness of the CpG sites we screened from a biological perspective. Finally, these key sites were used to develop diagnostic models for five age-related diseases, including breast cancer, kidney cancer, lung cancer, Alzheimer’s disease, and Parkinson’s disease.

### 4.2. Datasets

**Figure 17:**
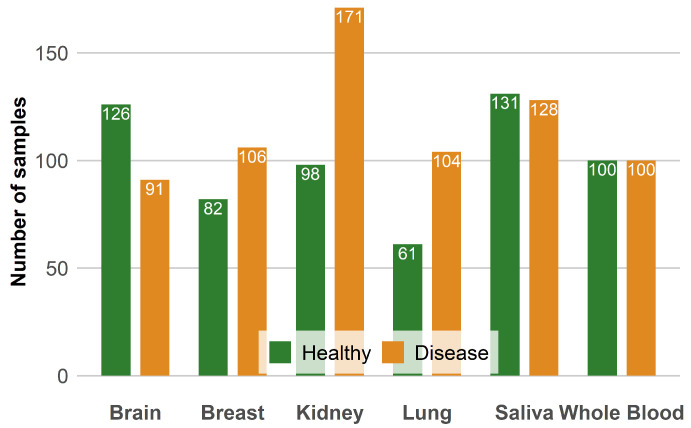
This figure shows the number of data we selected from each organization. The disease represents the data of the disease state, and Healthy represents the corresponding control group data. From this figure, we can see that the data we selected is reasonable.

This paper uses 12 public datasets from the Gene Expression Omnibus (GEO) database, which is established and maintained by the National Center for Biotechnology Information (NCBI). These datasets were generated using the Illumina Infinium 450k human DNA methylation microarray. In this study, we included samples from multiple tissues and biofluids, including kidney, saliva, brain, whole blood, breast, and lung tissues, totaling 1321 samples. We included tissue samples to more accurately screen key CpG sites for age-related diseases, which directly reflect the occurrence and development of the disease. Biofluid samples were included because they are easy to obtain, non-invasive, and have high clinical application potential. Biofluid samples can reflect the indirect effects of the disease, enrich the expression of disease characteristics, and help improve the broad applicability and robustness of diagnostic algorithms. By analyzing these samples from different tissue sources, we aim to reveal genetic variations that are unique to each tissue and understand the pathobiological mechanisms by which these variations lead to related degenerative diseases or cancers.The distribution of the data used in this study in each organization is shown in the Figure 17.

For kidney tissue, we selected three datasets: GSE52955, GSE59157, and GSE61441. GSE52955 involves genome-wide DNA methylation analysis of normal and tumor tissues from the urinary system (prostate, kidney, and bladder), from which we selected the kidney data. GSE59157 is designed to compare the epigenomic profiles of clear cell renal cell carcinoma (ccRCC) tissue with matched normal kidney tissue. GSE61441 contains genome-wide DNA methylation analysis of normal kidney tissue, nephrogenic quiescence, and Wilms’ tumor, and we selected the data for normal kidney tissue. For saliva samples, we used dataset GSE111223, which includes genome-wide DNA methylation analysis of saliva samples from 128 Parkinson’s disease (PD) patients and 131 control individuals. This dataset is designed to explore the epigenetic changes associated with PD. For brain samples, we selected samples with Alzheimer’s disease (AD) and their corresponding control samples, including two datasets: GSE80970 and GSE59685. GSE80970 consists of samples taken from the prefrontal cortex and superior temporal gyrus tissues of 147 individuals with varying degrees of AD pathology. GSE59685 involves cross-tissue methylome analysis of AD, with samples taken from multiple tissues of 122 donors. Genomic DNA was isolated from four brain regions in each donor: the entorhinal cortex (EC), superior temporal gyrus (STG), prefrontal cortex (PFC), and cerebellum (CER). For whole blood tissue, we selected the GSE72774, which contains 508 whole blood samples, including 289 from PD patients and 219 control samples.To ensure sample balance, we selected 100 PD samples and 100 control samples. For breast tissue, we used three datasets: GSE52865, GSE52270, and GSE60185. GSE52865 provides whole-genome DNA methylation analysis of normal and tumor breast tissues, including 40 primary breast tumors and 17 normal breast tissues. GSE52270 includes high-resolution DNA methylation array analysis of human cancer samples and normal control tissues, from which we selected the breast cancer samples. GSE60185 contains whole-genome DNA methylation maps of 285 breast tissue samples, which are divided into normal tissue samples and breast cancer samples. For lung tissue, we selected two datasets: GSE51077 and GSE63704. GSE51077 contains 36 samples, comprising whole-genome DNA methylation analysis of DNA samples obtained from normal lung tissue and lung cancer tissue. GSE63704 includes lung biopsy samples obtained via bronchoscopy, comprising 17 lung cancer patients, 37 idiopathic pulmonary fibrosis patients, 32 chronic obstructive pulmonary disease patients, and healthy lung samples with specific DNA methylation patterns. We selected lung cancer and healthy samples for our analysis.

To verify the generalization ability of our model and the accuracy of the screened sites, we conducted independent tests on four datasets: GSE111629, GSE66695, GSE75008, and GSE43414. GSE111629 contains whole-genome DNA methylation data from whole blood samples of Parkinson’s disease (PD) patients, including 335 PD patients and 237 controls. GSE66695 provides whole-genome DNA methylation analysis of normal and breast cancer samples, consisting of 40 normal samples and 80 breast cancer samples. GSE75008 contains DNA methylation data from lung tissue, including 40 normal lung tissue samples and 40 lung cancer samples. GSE43414 is a study focused on preprocessing methods for methylation array data and includes DNA methylation data from 695 samples across 11 different groups. From this dataset, we selected brain tissue samples, comprising 61 Alzheimer’s disease samples and 57 control samples.

This paper selected multiple datasets related to age-ralated diseases, covering different tissue types. Table 3 shows the relationship between each disease and its corresponding tissue type.

### 4.3. Screening of Key CPG Sites

This study used a two-stage screening approach to identify key CpG sites: first, the chi-square test was used to screen out CpG sites that were significantly associated with tissue specificity; then, logistic regression was used to further screen out sites associated with age-ralated diseases.

To avoid disease-specific effects on CpG sites, we exclusively selected healthy samples from our datasets. We have a total of m = 450 K CpG sites.

(1) $$D_{healthy}=\left\{X_{i},y_{i}|state_{i}=‘\mathrm{healthy}’\right\}$$

where $X_{i}=\left[C_{i1},C_{i2},\ldots ,C_{im}\right]$ is the feature vector of the *i*-th sample, representing the methylation degree of the CpG site in the range [0,1]. $y_{i}=\left[T_{1},T_{2},\ldots ,T_{6}\right]$ is the tissue of origin of the sample.

We selected CpG sites associated with tissue type by calculating the chi-square statistic and significance of the methylation degree of each CpG site and tissue type *y*.

(2) $$\chi^{2}\left(C_{j},y\right)=\sum_{k=1}^{m}\sum_{t=1}^{6}\frac{{\left(O_{kt}-E_{kt}\right)}^{2}}{E_{kt}}$$

(3) $$p-value=1-F_{\chi^{2}}\left(\chi^{2},df\right)$$

where $\chi^{2}$ is the Chi-square statistic, $F_{\chi^{2}}$ is the cumulative distribution function of the chi-square distribution, which indicates the probability that $\chi^{2}$ is less than or equal to a certain value. The df is the degrees of freedom for the chi-square test. The *p*-value represents the significance level of the observed $\chi^{2}$.

The smaller the *p*-value, the more significant the correlation between *C* and *y*. When it is less than 0.05, we believe that there is a significant correlation between $C_{j}$ and *y*. In this task, in order to strictly screen out tissue-specific CpG sites, we set the *p*-value threshold to 0.01. Therefore, the CpG sites we screened are as follows:

(4) $$\mathrm{Tissue}-\mathrm{Specificity}-\mathrm{CpGs}=\{j|p-value\left(C_{j},y\right)<\tau_{p}\}$$

where $\tau_{p}=0.01$ is the threshold of the significance level.

We screened m = 13,158 tissue-specific CpG sites in this way.

We further used logistic regression to analyze the sites identified by the chi-square test, aiming to extract age-ralated disease-specific CpG sites from these tissue-specific sites. Logistic regression is a machine learning algorithm for binary classification that predicts the probability of an outcome variable by linearly combining the predictor variables with their respective coefficients. We calculated the weights of these predictor variables to determine the degree of association between each CpG site and the age-ralated disease [38].

Our dataset is all samples, but only the tissue-specific CpG sites selected by the chi-square test are retained. It is represented as follows:

(5) $$D=\{\left(C_{i},y_{i},T_{i}|i=1,2,\ldots ,n\right)\}$$

(6) $$y_{i}=\left\{\begin{array}{c} 1\;\mathrm{disease}\\ 0\;\mathrm{healthy} \end{array}\right.$$

(7) $$D_{healthy}=\{\left(C_{i},y_{i},T_{i}|y_{i}=0\right\}$$

(8) $$D_{disease}=\{\left(C_{i},y_{i},T_{i}|y_{i}=1\right\}$$

(9) $$D=D_{healthy}\cup D_{disease}$$

where $C_{i}\in R^{2}$ is a feature vector of sample i containing m tissue-specific CpG sites, $y_{i}$ is health/disease labels for sample *i*.

We then screened the corresponding disease-specific CpG sites in *t* = 6 tissues.

(10) $$X_{T_{t}}=\left[C_{1},C_{2},\ldots ,C_{m}\right]$$

(11) $$P\left(y_{ti=1|X_{T_{i}}}\right)=\frac{1}{1+e^{-(\beta_{o}+C_{T_{i}}^{T}\beta )}}$$

(12) $$Rank\left(C_{j}\right)=argsort\left(\left|\beta \right|,\mathrm{descending}\right)$$

(13) $$Selected_{Features_{T_{t}}}=\{\left(C_{j}|j\in \mathrm{Top}400\mathrm{by}\left|\beta_{j}\right|\right\}$$

where $P(y_{ti}=1)$ is the probability that sample *i* belongs to the disease state, $\beta =[\beta_{1},\beta_{2},\ldots ,\beta_{m}]$ is a feature weight vector for the logistic regression model.

We selected CpG sites that exhibit both tissue specificity and disease specificity using the above method.

### 4.4. Diagnosis Model Construction

**Figure 18:**
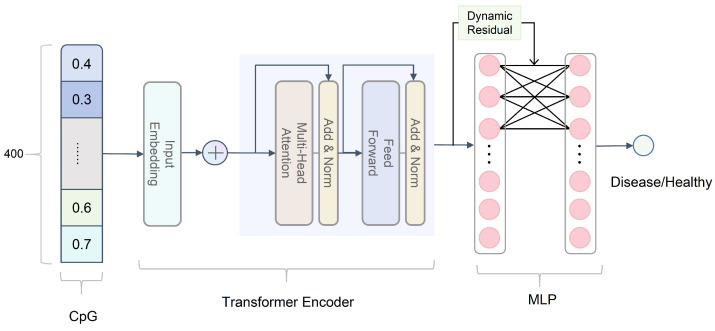
The Model structure. We convert the screened methylation sites into vectors through Transformer, and then diagnose the disease through MLP.

This study proposed a model based on a Transformer [39] and dynamic residual mechanism for disease diagnosis using the methylation level of CpG sites.The model aims to capture the complex nonlinear relationship between CpG sites and enhance the model’s ability to focus on key features through the dynamic residual mechanism, thereby achieving efficient and accurate disease classification. The disease diagnosis model we use is shown in the Figure 18.

Our input data is the methylation level of 400 CpG sites in a single sample, represented as a feature vector, $C=\left\{c_{1},c_{2},\ldots ,c_{i},\ldots ,c_{400}\right\}\in R^{d}$, Where $c_{i}$ represents the methylation level of the ith CpG site. To adapt to the Transformer input format, we expand the vector C in the channel dimension and transform it into $C^{\prime }=C\in R^{1\times d}$.

The Transformer encoder layer uses a multi-head self-attention mechanism to capture different dependencies and feature representations from the input sequence. We input vector $C^{\prime }$ into the Transformer encoder layer for feature extraction and obtain a feature vector containing the global dependencies between CpG sites.

(14) $$H=TransformerEncoder(C^{\prime })$$

where $H\in R^{1\times d}$ is the output feature of the Transformer encoder and also the input feature of the dynamic residual layer.It plays a connecting role in the model, connecting the global feature extraction of the Transformer encoder and the feature optimization of the dynamic residual layer.

We add a dynamic weight control mechanism based on the fully connected layer to adaptively adjust the retention and transformation of input features to more effectively capture complex feature representations. It improves the traditional residual connection by introducing a gating mechanism to dynamically adjust the weight of the residual path according to the input data. It consists of two parts: fully connected feature transformation and gating mechanism.

First, we use the fully connected layer to perform a linear transformation on the input to extract deeper features. Then we introduce a gating structure to dynamically calculate the weights of the feature transformation and the residual path, and generate the weight coefficients through the Sigmoid function. The formula is as follows:

(15) $$T=FC\left(H\right)=HW_{fc}+b_{fc}$$

(16) $$G=\sigma (HW_{gate}+b_{gate})$$

where $W_{fc},W_{gate}\in R^{d\times d}$ are the weight matrices of the fully connected layer and the gating layer, respectively. G is a gating weight used to dynamically adjust the fusion ratio of the input feature *H* and the feature transformation result *T*. It is the core mechanism of the dynamic residual layer. By generating weights according to the input data, it flexibly controls the proportion of the residual path and the feature transformation path. $b_{fc}\in R^{d}$ is the bias term that appears in the feature transformation of the dynamic residual layer. It is used to adjust the baseline value of the linear transformation output, giving the model greater flexibility and learning ability. $b_{gate}\in R^{d}$ is the bias term of the gating mechanism in the dynamic residual layer, which is used to adjust the value range of the dynamic weight *G*, thereby affecting the fusion ratio of the residual path and feature transformation.

We then use the weighted sum of the feature transformation and residual paths to generate the final output.

(17) O=G·T+(1−G)·H

where $O\in R^{d}$ is the output of the dynamic residual layer.

We pass the output of the dynamic residual layer through the fully connected layer to complete the final disease diagnosis task.

(18) $$y=Softmax(OW_{class}+b_{class})$$

where $W_{class}\in R^{d\times 2}$ are the classifier weights. $y\in R^{2}$ is the probability distribution over the classes.

Through the above formula description, we show that the model extracts and optimizes the input 400 CpG site features C and finally completes the disease diagnosis task.

## 5. Conclusions

This paper uses a combination of chi-square tests and logistic regression to screen out CpG sites that are both tissue-specific and disease-specific and uses our proposed age-related disease diagnosis model to fit these sites, which can effectively diagnose these diseases in various tissues and achieve significant results. These findings illustrate the potential application of DNA methylation tissue specificity and deep learning models in medical diagnosis.

In terms of GO and KEGG enrichment analysis of the screened sites, the study revealed multiple biological processes and metabolic pathways related to organ function and disease progression, indicating that our diagnostic method is scientifically based.

Although our proposed diagnostic method has shown good results in most tissues, there is still room for improvement in saliva datasets, and the generalization ability in blood tissues also needs to be further strengthened. In the future, we will optimize the model, better process data, and improve our disease diagnosis model to provide better diagnostic tools for the clinical field.

## Figures and Tables

**Table 1:** Changes of some of the selected methylation sites between disease and health.

Tissue	CpG_Site	H_M	D_M	Magnification	H_SE	D_SE
Breast	cg16732616	0.039	0.4305	11	0.006	0.016
	cg14038391	0.04	0.399	9.975	0.006	0.018
	cg16781647	0.054	0.3825	7.08	0.006	0.020
	cg10136354	0.0565	0.393	6.95	0.006	0.017
	cg06616729	0.0755	0.417	5.52	0.005	0.016
Saliva	cg04944784	0.169	0.2935	1.74	0.018	0.016
	cg06048169	0.653	0.4225	0.647	0.020	0.020
	cg17842918	0.087	0.0655	0.753	0.012	0.013
	cg22633036	0.252	0.313	1.242	0.012	0.013
	cg15922174	0.137	0.1045	0.763	0.010	0.009
Lung	cg23154059	0.19	0.3385	1.782	0.015	0.013
	cg02155658	0.19	0.317	1.668	0.012	0.012
	cg05157171	0.106	0.154	1.448	0.007	0.011
	cg08454053	0.163	0.236	1.448	0.008	0.010
	cg01465169	0.246	0.1445	0.587	0.018	0.015
Whole Blood	cg23627948	0.169	0.3035	1.791	0.017	0.016
	cg09703840	0.568	0.7955	1.401	0.026	0.022
	cg12575883	0.2415	0.1475	0.611	0.012	0.011
	cg11231949	0.086	0.118	1.372	0.009	0.010
	cg13532885	0.608	0.384	0.631	0.017	0.018
Brain	cg03440272	0.242	0.33	1.364	0.014	0.014
	cg19797896	0.443	0.578	1.305	0.015	0.015
	cg22904711	0.167	0.212	1.269	0.006	0.004
	cg24648384	0.272	0.344	1.265	0.010	0.009
	cg08373528	0.557	0.466	0.837	0.002	0.003
Kidney	cg16507965	0.162	0.21	1.296	0.008	0.007
	cg09703840	0.5	0.634	1.268	0.019	0.012
	cg24648384	0.272	0.344	1.265	0.010	0.009
	cg05522042	0.782	0.581	0.743	0.016	0.016
	cg15033013	0.686	0.738	1.076	0.001	0.002

**Table 2:** Gene table corresponding to some of the selected methylation sites.

Tissue	CpG_Site	Gene
Breast	cg16732616	DMRTA2
	cg14038391	—
	cg16781647	EMX1
	cg10136354	EMX1
	cg06616729	AC091076.1
Saliva	cg04944784	GAREM2
	cg06048169	PKD1L2
	cg17842918	ATP11A
	cg22633036	FGFR2
	cg15922174	CRB2
Lung	cg23154059	WNT6
	cg02155658	PAX1
	cg05157171	AL671277.1; HLA-A
	cg08454053	AC099684.2; RTN4RL1
	cg01465169	ABHD15-AS1; CORO6
Whole Blood	cg23627948	AMZ1
	cg09703840	—
	cg12575883	COX19
	cg11231949	AC023095.1; NKAIN3
	cg13532885	SYN1; Z84466.1
Brain	cg03440272	AC233976.1; AL158055.1
	cg19797896	AC027644.4; RABGEF1
	cg22904711	KCNN4
	cg24648384	AMZ1; GNA12
	cg08373528	PRPH2
Kidney	cg16507965	ARX
	cg09703840	—
	cg24648384	AMZ1; GNA12
	cg05522042	KIAA0513
	cg15033013	PRKCZ

**Table 3:** Tissue types for each disease.

Tissue	Disease
Brain	Alzheimer’s
Saliva	Parkinson’s
Whole Blood	Parkinson’s
Kidney	Kidney Cancer
Lung	Lung Cancer
Breast	Breast Cancer

## Data Availability

All DNA methylation datasets used in this paper are from the Gene Expression Omnibus (GEO) database, which is a public comprehensive repository of gene expression data. All datasets are available at the following website: https://www.ncbi.nlm.nih.gov/geo/ (accessed on 1 September 2024).

## Supplementary Materials

The following supporting information can be downloaded at: https://www.mdpi.com/article/10.3390/ijms26020452/s1.

## References

[1] Chang A.Y., Skirbekk V.F., Tyrovolas S., Kassebaum N.J., Dieleman J.L. (2019). Measuring population ageing: An analysis of the Global Burden of Disease Study 2017. Lancet Public Health.

[2] Self W.K., Holtzman D.M. (2023). Emerging diagnostics and therapeutics for Alzheimer disease. Nat. Med..

[3] Dauer W., Przedborski S. (2003). Parkinson’s disease: Mechanisms and models. Neuron.

[4] Johnson A.A., Akman K., Calimport S.R., Wuttke D., Stolzing A., De Magalhaes J.P. (2012). The role of DNA methylation in aging, rejuvenation, and age-related disease. Rejuvenation Res..

[5] Moore L.D., Le T., Fan G. (2013). DNA methylation and its basic function. Neuropsychopharmacology.

[6] Song F., Smith J.F., Kimura M.T., Morrow A.D., Matsuyama T., Nagase H., Held W.A. (2005). Association of tissue-specific differentially methylated regions (TDMs) with differential gene expression. Proc. Natl. Acad. Sci. USA.

[7] Thompson R.F., Atzmon G., Gheorghe C., Liang H.Q., Lowes C., Greally J.M., Barzilai N. (2010). Tissue-specific dysregulation of DNA methylation in aging. Aging Cell.

[8] Chen Y., Breeze C.E., Zhen S., Beck S., Teschendorff A.E. (2016). Tissue-independent and tissue-specific patterns of DNA methylation alteration in cancer. Epigenetics Chromatin.

[9] Miller R.H., Pollard C.A., Brogaard K.R., Olson A.C., Barney R.C., Lipshultz L.I., Johnstone E.B., Ibrahim Y.O., Hotaling J.M., Schisterman E.F. (2023). Tissue-specific DNA methylation variability and its potential clinical value. Front. Genet..

[10] Karaglani M., Panagopoulou M., Baltsavia I., Apalaki P., Theodosiou T., Iliopoulos I., Tsamardinos I., Chatzaki E. (2022). Tissue-specific methylation biosignatures for monitoring diseases: An in silico approach. Int. J. Mol. Sci..

[11] Park C., Ha J., Park S. (2020). Prediction of Alzheimer’s disease based on deep neural network by integrating gene expression and DNA methylation dataset. Expert Syst. Appl..

[12] Liu B., Liu Y., Pan X., Li M., Yang S., Li S.C. (2019). DNA methylation markers for pan-cancer prediction by deep learning. Genes.

[13] Ferrer I., Andrés-Benito P., Ausín K., Pamplona R., Del Rio J.A., Fernández-Irigoyen J., Santamaría E. (2021). Dysregulated protein phosphorylation: A determining condition in the continuum of brain aging and Alzheimer’s disease. Brain Pathol..

[14] Stankiewicz T.R. (2014). Rho GTPases in Neuronal Apoptosis and Neurodegeneration. Ph.D. Thesis.

[15] Rajaei S., Karima S., Sepasi Tehrani H., Shateri S., Mahmoodi Baram S., Mahdavi M., Mokhtari F., Alimohammadi A., Tafakhori A., Amiri A. (2020). Conformational change and GTPase activity of human tubulin: A comparative study on Alzheimer’s disease and healthy brain. J. Neurochem..

[16] Mühle C., Bilbao Canalejas R.D., Kornhuber J. (2019). Sphingomyelin synthases in neuropsychiatric health and disease. Neurosignals.

[17] Wei X., Huang G., Liu J., Ge J., Zhang W., Mei Z. (2023). An update on the role of Hippo signaling pathway in ischemia-associated central nervous system diseases. Biomed. Pharmacother..

[18] Wang Y., Zhou C.J., Liu Y. (2018). Wnt signaling in kidney development and disease. Prog. Mol. Biol. Transl. Sci..

[19] Satardey R., Yadav R., Das M., Pal D.K. (2022). Analysis of expression pattern of proteins associated with AKT/mTOR signaling pathway in kidney cancer development. Ann. Med. Sci. Res..

[20] Huang D., Ding Y., Luo W.M., Bender S., Qian C.N., Kort E., Zhang Z.F., VandenBeldt K., Duesbery N.S., Resau J.H. (2008). Inhibition of MAPK kinase signaling pathways suppressed renal cell carcinoma growth and angiogenesis in vivo. Cancer Res..

[21] Cinar B., Alp E., Al-Mathkour M., Boston A., Dwead A., Khazaw K., Gregory A. (2021). The Hippo pathway: An emerging role in urologic cancers. Am. J. Clin. Exp. Urol..

[22] Solarek W., Koper M., Lewicki S., Szczylik C., Czarnecka A.M. (2019). Insulin and insulin-like growth factors act as renal cell cancer intratumoral regulators. J. Cell Commun. Signal..

[23] Figura M., Sitkiewicz E., Świderska B., Milanowski Ł., Szlufik S., Koziorowski D., Friedman A. (2021). Proteomic profile of saliva in Parkinson’s disease patients: A proof of concept study. Brain Sci..

[24] Ma K.Y., Fokkens M.R., Reggiori F., Mari M., Verbeek D.S. (2021). Parkinson’s disease–associated VPS35 mutant reduces mitochondrial membrane potential and impairs PINK1/Parkin-mediated mitophagy. Transl. Neurodegener..

[25] Corti O., Hampe C., Koutnikova H., Darios F., Jacquier S., Prigent A., Robinson J.C., Pradier L., Ruberg M., Mirande M. (2003). The p38 subunit of the aminoacyl-tRNA synthetase complex is a Parkin substrate: Linking protein biosynthesis and neurodegeneration. Hum. Mol. Genet..

[26] Stieglitz K.A. (2018). Structural insights for drugs developed for phospholipase D enzymes. Curr. Drug Discov. Technol..

[27] Stern S., Lau S., Manole A., Rosh I., Percia M.M., Ben Ezer R., Shokhirev M.N., Qiu F., Schafer S., Mansour A.A. (2022). Reduced synaptic activity and dysregulated extracellular matrix pathways in midbrain neurons from Parkinson’s disease patients. Npj Park. Dis..

[28] Qadri R., Namdeo M., Behari M., Goyal V., Sharma S., Mukhopadhyay A.K. (2018). Alterations in mitochondrial membrane potential in peripheral blood mononuclear cells in Parkinson’s disease: Potential for a novel biomarker. Restor. Neurol. Neurosci..

[29] Kong Y., Liang X., Liu L., Zhang D., Wan C., Gan Z., Yuan L. (2015). High throughput sequencing identifies microRNAs mediating *α*-synuclein toxicity by targeting neuroactive-ligand receptor interaction pathway in early stage of drosophila Parkinson’s disease model. PLoS ONE.

[30] Tang S.C., Wu M.F., Wong R.H., Liu Y.F., Tang L.C., Lai C.H., Hsu C.P., Ko J.L. (2011). Epigenetic mechanisms for silencing glutathione S-transferase m2 expression by hypermethylated specificity protein 1 binding in lung cancer. Cancer.

[31] Li Y., Lin M., Wang S., Cao B., Li C., Li G. (2022). Novel angiogenic regulators and anti-angiogenesis drugs targeting angiogenesis signaling pathways: Perspectives for targeting angiogenesis in lung cancer. Front. Oncol..

[32] Wigén J., Elowsson-Rendin L., Karlsson L., Tykesson E., Westergren-Thorsson G. (2019). Glycosaminoglycans: A link between development and regeneration in the lung. Stem Cells Dev..

[33] Krause N., Wegner A. (2020). Fructose metabolism in cancer. Cells.

[34] Menashe I., Maeder D., Garcia-Closas M., Figueroa J.D., Bhattacharjee S., Rotunno M., Kraft P., Hunter D.J., Chanock S.J., Rosenberg P.S. (2010). Pathway analysis of breast cancer genome-wide association study highlights three pathways and one canonical signaling cascade. Cancer Res..

[35] Sun Z., Jiang Q., Gao B., Zhang X., Bu L., Wang L., Lin Y., Xie W., Li J., Guo J. (2023). AKT blocks SIK1-mediated repression of STAT3 to promote breast tumorigenesis. Cancer Res..

[36] Herzog E.M., Eggink A.J., Willemsen S.P., Slieker R.C., Felix J.F., Stubbs A.P., van der Spek P.J., van Meurs J.B., Heijmans B.T., Steegers-Theunissen R.P. (2021). The tissue-specific aspect of genome-wide DNA methylation in newborn and placental tissues: Implications for epigenetic epidemiologic studies. J. Dev. Orig. Health Dis..

[37] Wang Y.Z., Xu W.W., Zhu D.Y., Zhang N., Wang Y.L., Ding M., Xie X.M., Sun L.L., Wang X.X. (2018). Specific expression network analysis of diabetic nephropathy kidney tissue revealed key methylated sites. J. Cell. Physiol..

[38] Saeys Y., Inza I., Larranaga P. (2007). A review of feature selection techniques in bioinformatics. Bioinformatics.

[39] Vaswani A. (2017). Attention is all you need. Adv. Neural Inf. Process. Syst..

